# Encapsulation of Basil Essential Oil by Paste Method and Combined Application with Mechanical Trap for Oriental Fruit Fly Control

**DOI:** 10.3390/insects12070633

**Published:** 2021-07-13

**Authors:** Tibet Tangpao, Patcharin Krutmuang, Wilawan Kumpoun, Pensak Jantrawut, Tonapha Pusadee, Ratchadawan Cheewangkoon, Sarana Rose Sommano, Bajaree Chuttong

**Affiliations:** 1 Plant Bioactive Compound Laboratory, Faculty of Agriculture, Chiang Mai University, Chiang Mai 50200, Thailand; tibet_tangpao@cmu.ac.th; 2 Department of Plant and Soil Sciences, Faculty of Agriculture, Chiang Mai University, Chiang Mai 50200, Thailand; tonapha.p@cmu.ac.th; 3 Department of Entomology and Plant Pathology, Faculty of Agriculture, Chiang Mai University, Chiang Mai 50200, Thailand; patcharin.k@cmu.ac.th (P.K.); ratchadawan.c@cmu.ac.th (R.C.); 4 Innovative Agriculture Research Center, Faculty of Agriculture, Chiang Mai University, Chiang Mai 50200, Thailand; 5 Science and Technology Research Institute, Chiang Mai University, Chiang Mai 50200, Thailand; wilawan.k@cmu.ac.th; 6 Department of Pharmaceutical Sciences, Faculty of Pharmacy, Chiang Mai University, Chiang Mai 50200, Thailand; pensak.j@cmu.ac.th

**Keywords:** behaviour, bottle trap, holy basil, integrated pest management, phenylpropanoids, retaining

## Abstract

**Simple Summary:**

Essential oils of the *Ocimum* spp. comprised of the volatile phenylpropanoids known for the Oriental fruit fly attractant property. However, fully exposing the essential oils in the field limited their ability. Therefore, we proposed a cheap yet effective paste encapsulation technique combined with a plastic trap for controlling tropical fruit fly in tropical fruit orchard.

**Abstract:**

In this work, the chemical compositions of basils oils, including those of lemon basil, white holy basil, Thai basil, tree basil and red holy basil, were analysed. Methyl eugenol was detected in all types of basils. The essential oils of red and white holy basils possessed a comparable ability (~25%) to attract male Oriental fruit fly to the synthesised fruit fly attractant in the laboratory experiment. To control the release of the active ingredients, the white holly basil oil (WBO) was encapsulated with maltodextrin (MD) and gum arabic (GA) by paste method. The essential oil is retained in the wall complex much longer with the addition of MD. The results also revealed that the combination of the MD:GA (25:75) had the highest loading efficiency of the oil (9.40%) as observed by the numerous porous structures by scanning electron microscopy. Fourier-transform infrared spectra of the encapsulated polymer confirmed traces of essential oil functional groups. The field test study advised that WBO-encapsulated products improved fruit fly attractive efficiency by maintaining the release rate of basil essential oil.

## 1. Introduction

Tropical fruit production along with postharvest handling chains, particularly those of soft skin types, such as avocado, mango and papaya, have been severely desecrating by the invasion of Oriental fruit fly (ORF; *Bactrocera dorsalis* Hendel). In Thailand, for instance, the estimated loss from this pest alone is roughly over 100-million US$ per year, and the most vulnerable crop being mango [1,2]. The infestation leads to a vast reduction of the consumption demand and has raised quarantine restrictions of the importing countries. Many strategies have been used to control the population of the ORF, for example, spraying with insecticide or protein bait, trapping with attractants, releasing of natural enemies, the Sterile Insect Technique (SIT), as well as the combined approach in the Integrated Pest Management (IPM) [2,3]. While the SIT-integrated management scheme appeared to be effective, the most popular methods are insecticide cover spray and bait spraying [4,5]. However, the remaining pesticide risks the health and welfare of both farmers and consumers and, more importantly, leaves severe damage to the environment [6,7]. The male-lure-based trapping system is among the interests for fruit fly bio-control approaches. Trends are moving toward incorporating the trap with natural plant products for more sustainable practices [8,9,10]. More importantly, a plant-based active ingredient is also economical, non-hazardous, environment-friendly, and simple technology. It is also insect-genera specific, which is effective as a bio-control agent [11,12].

Plant phenylpropanoids of Volatile Organic Compounds (VOCs) are derived from the shikimate secondary metabolite biosynthesis pathway between L-phenylalanine and L-tyrosine [13]. Interestingly, the volatiles of these types serve as natural attractants to numerous species of Dacine fruit flies (Tephritidae: Dacinae: Dacini) [14]. Among those, methyl eugenol is highly attractive to male ORFs [8,9,15]. It is also known as a precursor or booster of male ORF’s sex pheromone [16]. Methyl eugenol has been used in the IPM to control ORF population [17,18]. However, as many synthesised controlling substances are banned from organic fruit production, VOCs from natural sources have increasingly gained attention lately [8,9,10]. This compound is a widely distributed natural plant product and consisted of the essential oils from as many as 200 tropical plant species such as anise (*Pimpinella anisum*), nutmeg (*Myristica fragrans*), and the *Ocimum* spp. [7,19,20,21,22,23]. The genus *Ocimum* of the Lamiaceae is recognised globally as the wealthiest essential oil-bearing family comprising more than 150 species grown widely throughout the tropical and temperate regions [24]. They are collectively known as “basils” of landscape, culinary and medicinal importance [25]. In Asia and the Pacific regions, four basil varieties viz., holy basil (*O. sanctum*), lemon basil (*O. citriodorum*), sweet or Thai basil (*O. basilicum*), and tree basil (*O. gratissimum*) were commonly grown in large quantities for culinary purposes [26,27]. Plants of the *Ocimum* spp. have been cultivated for the essential oil extraction industry, especially the sweet basil (*O. basilicum* L.), in which the annual production was ca. 50 tonnes globally [28,29]. The essential oils are, indeed, of high demand as flavouring agents, cosmetic ingredients and medicines [30,31,32,33]. In 2018, Tangpao et al. [34] illustrated that estragole, eugenol, and methyl eugenol were among the significant VOCs found in the essential oils of some Thai *Ocimum* spp. plants. Thus, it has been used for the controlling of agricultural pests such as common fruit flies by attracting and killing ability [24,35,36,37]. Accordingly, it is interesting to apply the essential oil from basils as a natural lure for ORF. 

There are, however, only limited studies on the use of basil essential oils as ORF biocontrol along with their chemotypes relations. However, the constraint is that essential oil generally decomposes fast when exposed to outdoor conditions [38]. Encapsulation can retain VOCs through physical or chemical interaction with matrix complexation, which improves stability and controls the release [39]. The most used and cheap technique is dispersing the essential oil onto the matrix of wall materials known as paste method complexation. With this technique, the complexation is created by kneading the essential oil with a saccharide paste containing 10–40% water, and no organic solvent is required [40]. To the best of our knowledge, there is no research conducting the efficacy of essential oils from locally available basil species to attract the ORF as well as the development of relating innovative products. The aims of this study are to determine the chemical compositions of basil essential oils from the commonly available Thai *Ocimum* spp. and to examine their efficiency as a lure for controlling the ORF. Lastly, we also propose an alternative product formulation with a simple encapsulating technique. This research is also one of the first studies to apprise paste encapsulation technology for crop protection and pest management.

## 2. Materials and Methods

### 2.1. Chemicals

Dichloromethane (RCI Labscan, Bangkok, Thailand), toluene (RCI Labscan, Bangkok, Thailand), acetone (RCI Labscan, Bangkok, Thailand), alkane standard solution C_8_-C_20_ (Fluka^®^ Analytical, Munich, Germany), commercial fruit fly attractant (methyl eugenol) (S.S.U. Intertrade, Bangkok, Thailand), maltodextrin DE10 (Krungthepchemi, Bangkok, Thailand), and gum arabic (Krungthepchemi, Bangkok, Thailand). 

### 2.2. Insects

ORF pupae were obtained from Plant Protection Research and Development, Department of Agriculture, Bangkok, Thailand. The adult ORFs were fed daily with an artificial diet (yeast extract and sugar with 1:3 ratio) and water (wet sponge). ORFs were between 15–30-day-old (days after emerging from pupae) upon the attractiveness laboratory testing.

**Experiment 1: Volatile analysis of the essential oils and *B. dorsalis* attractiveness**

### 2.3. Essential Oil Extraction

The cut stems of *Ocimum* spp. plants, including *O. sanctum* var. Rama (red holy basil; RB), *O. sanctum* var. Shyama (white holy basil; WB)*, O. citriodorum* (lemon basil; LB), *O. basilicum* var. thyrsiflorum (Thai basil; TB), and *O. gratissimum* (tree basil; TrB), were collected fresh from the local contract farmer and brought to the laboratory immediately. The plant species and types were confirmed by their taxonomy [34]. The leaves were separated from the stems and shade-dried at room temperature (27–32 °C) until a constant weight was reached. Dried leaves (~300 g) were used for essential oil extraction in a 5 L Clevenger hydro-distillation apparatus containing 2.5 L of distilled water. The extraction was for 2 h at 120 °C. After cooling down at room temperature, the essential oil was removed and treated with anhydrous sodium sulphate to eliminate the remaining water [41].

### 2.4. Volatile Analyses

Gas Chromatography-Mass Spectrometry (GC-MS) analyses were accomplished with a Bruker-scion 436 GC-MS equipped with 30 m × 0.25 mm, Rxi-5Sil MS column (Restek, Bellefonte, PA, USA). Essential oil (2 µL at a dilution of 1% (*v/v*) in dichloromethane with a presence of 0.015 mg/mL toluene as an internal standard) was injected in a split mode (1:20). The oven temperature was set at 60 °C for 3 min and increased by 3 °C/min until 240 °C, where it was held for 6 min. The carrier gas was helium with a flow rate of 1 mL /min [34]. The interface with MS was at 200 °C, and mass spectra were taken at 70 eV in electron impact ionisation mode, with a scanning speed of 0.5 scans/s from *m/z* 20–350 [42]. The identification of the volatile compositions was by comparison with mass spectra in NIST 05.L and NIST 98.L libraries. The amount in µg/mL of essential oil was calculated relative to the amount of the internal standard.

### 2.5. Attractiveness

**Scheme 1:**
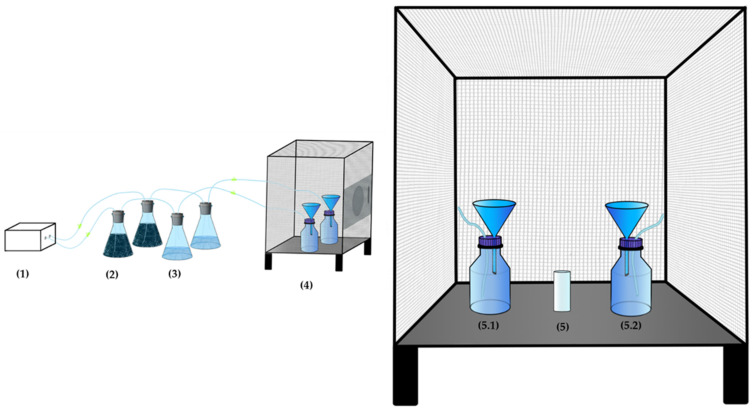
The attractiveness experiment using the two bottle-trap (two-choice olfactometer): (**1**) electric air pump, (**2**) activated charcoal flasks, (**3**) distilled water flasks, (**4**) experimental cage, (**5**) insects releasing point, and (**5.1**) and (**5.2**) bottle traps.

Attractiveness was performed in a mesh cage (50 × 40 × 40 cm) in a controlled room (23–25 °C; RH 40–70%) as modified from Biasazin et al. [43]. Two bottle-trap was placed inside the cage. The air was pumped in and filtered through the activated charcoal and distilled water then essential oil vapour was released through the top of the cone on bottle traps (Scheme 1). The airflow was adjusted to 0.50 L/min constantly, and all pieces of the experimental apparatus were joined by Teflon tubing. The concentration of the test solutions was 1% (*v/v*) of essential oil in acetone and acetone as a control solution. The test solution 10 µL was applied to 2 cm^2^ of filter paper 5 min before testing. One hundred ORFs (50 males and 50 females) were released between two bottles (control and treatment). The number of ORFs trapped in the bottle was recorded (male and female) after ORFs were released for 1 h. The bottles containing the test material were replaced and cleaned every single test. Four replications were determined for each test solution.

### 2.6. Insect Olfactory-Chemosensory Responses 

To investigate insect behaviour response to the essential oils, male and female flies were mounted alive with glue in the position that their frontal parts (heads, wings, and forelegs) could move freely. A 20 µL drop of the 1% essential oils or ethanol as the control was pipetted onto the filter paper located ~10 cm underneath the ORF approximately every 30–60 secs apart. The movement of the antennae and the forelegs were observed [44].

**Experiment 2: Paste matrix complexation with basil essential oil encapsulation**

The highly effective essential oil type was chosen to conduct this experiment. 

### 2.7. Paste Matrix Complexation

Wall materials were made according to Shrestha, Ho, and Bhandari [40], Bhandari et al. [45], and Carneiro et al. [46]. In the preparation, the mixture of the wall material (Table 1) was mixed with distilled water (40 °C) and stirred until completely dissolved. After that, the essential oil was added to the mixture solution at a concentration of 20% to total solids (wall materials). Emulsions were formed using a homogeniser operating at 18,000 rpm for 5 min, and the emulsion characteristic was recorded. 

Emulsion viscosity and stability were analysed according to Carneiro, Tonon, Grosso, and Hubinger [46]. Immediately after emulsion, the viscosity of the paste solution was measured by determining steady-shear flow curves using a Rheometer. Emulsion stability was analysed using 25 mL-aliquots of each sample transferred to graduated cylinders of 25 mL, sealed, stored at room temperature for one day, and the upper phase’s volume measured after 24 h. The stability was measured by the percentage of separation and expressed as:

Separation (%) = (H_1_/H_0_) × 100

where H_0_ represents the initial emulsion height, and H_1_ is the upper phase height.

### 2.8. Encapsulation Characteristics

The emulsion paste was dried using a vacuum oven at 60 °C for 4 h and then ground to a powder using a grinder. The encapsulation efficiency was quantitatively determined by a UV-visible spectrophotometer [47]. The capsule morphology was examined using Scanning Electron Microscopy (SEM). The capsule powder was mounted on double-sided tape attached to the specimen stub and then subjected to metallisation (sputtering) with a thin layer of gold. The images were viewed at magnifications of ×2000 using SEM (JEOL JSM-5910, Japan Electron Optics Laboratory Co., Ltd., Tokyo, Japan) with an accelerating voltage of 5 kV.

### 2.9. Fourier-Transform Infrared Spectroscopy (FTIR)

FTIR analysis was conducted using a DairyGuard^TM^ Milk Powder Analyser (PerkinElmer). Each sample was scanned by placing the sample on the ATR diamond crystal and applying the pressure tower. The spectrum was verified in the transparent mode from 450–4000 cm^−1^. 

### 2.10. Controlled Release of the Capsules

One gram of each capsule was placed into the 3 mL vial without sealing. The vials were then left at 25–30 °C (capsules were strictly maintained in dry condition), and weight loss was monitored in an analytical balance as a function of time for 3 h. The percentage of release was calculated in relation to the initial mass of the products [48].

**Experiment 3: Product efficacy in the field test study**

### 2.11. The Experiment Site

The experiment was conducted in a multi-crop orchard (Dusanee orchard) located in Mae Taeng District, Chiang Mai, Thailand (19°5′51.08″, 98°53′55.54″) in which mango and santol were cultivated 3 × 3 m alternately to one another. The test experiment was conducted during the peak harvesting of mango var. nam dok mai. The maintenance of the orchard was in accordant with Good Agricultural Practice (GAP). The orchard was in a healthy condition comprising a compact load of mango trees (five-year-old, small–medium tree size of ~3 m tall).

### 2.12. Trap

**Scheme 2:**
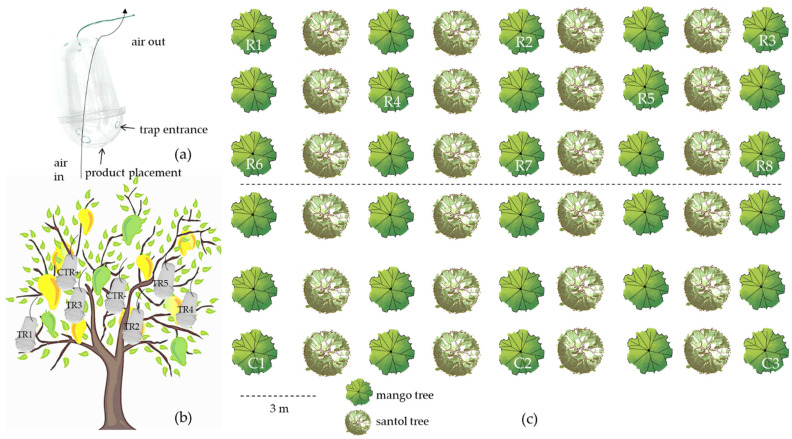
Field study of the Oriental fruit fly traps with encapsulated products during mango var. nam dok mai harvesting season. (**a**) Trap and (**b**) Trap hanging inside the canopy; TR1 = MD100, TR2 = GA100, TR3 = MD50GA50, TR4 = MD75GA25, TR5 = MD25GA75, CTR+ = white holy basil essential oil, and CTR- = no test solution (**c**) Experimental arrangement; R1–R8 as trap replications 1–8, and C1–C3 as traps with the commercial fruit fly attractant 1–3.

The trap was made from a plastic coffee cup (250 mL) with a round lid. Six holes were drilled on the cup (3 × Ø 1 cm on the lid and 3 × Ø 0.3 cm on the cup) (Scheme 2a). The capsule (1 g) was placed inside the lid, capped with the cup, and immediately tided onto the mango tree by upside-down position. The traps were hung up-side-down where natural air was able to flow naturally through holes. The efficacy of the encapsulated products was compared by placing traps inside the canopy at the height of ca 1.5–2 m (Scheme 2b). Insects caught on or in the traps were generally counted between 2.30–5.00 pm for 30, 60, 90, and 120 min, and thereafter 24, 48, and 72 h. A completely randomised design (CRD) was used, with eight replicates (Scheme 2c) [49]. The controls were the essential oil (5 µL) on a cotton ball and trap without the product. The product efficiency test was compared with traps with 5 µL on a cotton ball of the commercial fruit fly attractant. They were placed two rows away from the experiment. 

### 2.13. Statistical Analysis

The averages of all the experiments’ data were subjected to analysis of variance (ANOVA) at the significance level *p* = 0.05 using the IBM^®^ SPSS^®^ Statistics version 23. In addition, principal component analysis (PCA) to summarise graphical differences of volatile components of the essential oil was adopted using Minitab Statistical Software (Minitab^®^).

## 3. Results

**Experiment 1: Volatile analysis of the essential oils and *B. dorsalis* attractiveness (in-vitro study)**

### 3.1. Chemical Compositions

**Figure 1:**
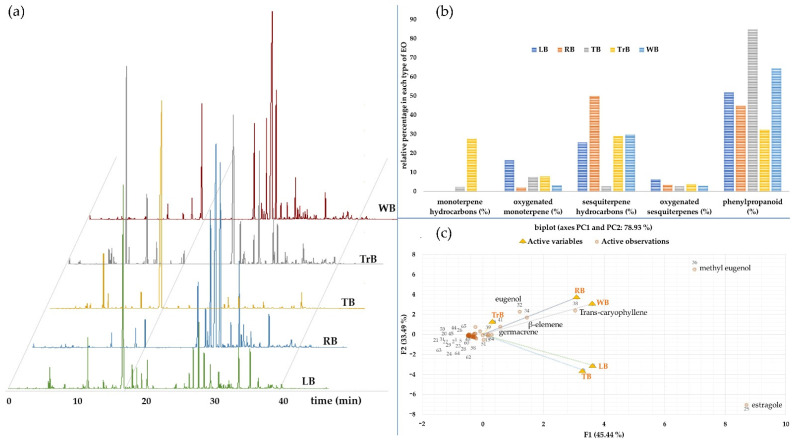
The chromatograms of the VOC profiles of different basils essential oils (LB; lemon basil (*O. citriodorum*), RB; red holy basil (*O. sanctum* var. Rama), TB; Thai basil (*O. basilicum* var. thyrsiflorum), TrB; tree basil (*O. gratissimum*), and WB; white holy basil (*O. sanctum* var. Shyama)) with the detailed analysis of the chemical compositions as illustrated in Table 2 (**a**), their relative percentage composition of volatile types (**b**), and a biplot diagram illustrating the relationships among the volatiles components with five basil types (**c**).

The VOCs and the relative amount of essential oil from five basils are illustrated in Table 2. Overall, a total of 65 volatile compounds was identified, by which estragole, eugenol, and methyl eugenol of the phenylpropanoids were dominant, followed by the sesquiterpenes (viz., trans-caryophyllene, trans-α-bergamotene, τ-cadinol, cis-α-bisabolene, β-elemene, and germacrene) and monoterpenes (viz., trans-ocimene, linalool, 1,8-cineole, and camphor). The primary constituents of LB essential oil were estragole (~316.87 µg/mL EO), trans-caryophyllene (~51.20 µg/mL EO), cis-α-bisabolene (~37.24 µg/mL EO), and linalool (~32.64 µg/mL EO). Red holy basil oil comprised of the mixture of 17 volatile compounds, of which it was dominated by methyl eugenol (~335.58 µg/mL EO) and trans-caryophyllene (~193.96 µg/mL EO), β-elemene (~119.06 µg/mL EO), and germacrene (~60.35 µg/mL EO). In the essential oil of TB, estragole was the highest (~609.47 µg/mL EO), followed by 1,8-cineole (~37.91 µg/mL EO), τ-cadinol (~14.08 µg/mL EO), and camphor (~13.57 µg/mL EO). As many as 35 volatile compositions were identified in the TrB oil, with eugenol (~224.24 µg/mL EO), trans-ocimene (~169.23 µg/mL EO), trans-α-bergamotene (~83.64 µg/mL EO), and linalool (~45.01 µg/mL EO) being the major components. White holy basil oil contained 23 compounds, including methyl eugenol (~372.57 µg/mL EO), trans-caryophyllene (~99.38 µg/mL EO), estragole (~84.17 µg/mLEO), and eugenol (~83.04 µg/mL EO). The difference in VOC profiles of each type of essential oil was displayed on the chromatograms, as illustrated in Figure 1a. 

The relationship of the VOCs and types of basil essential oil were elucidated using principal component analysis in Figure 1c. The biplots analysis depicted a total of 78.93% across the PCA range, which accounted for 45.44% in PC1 and 33.49% in PC2. The volatile compositions of all essential oil were similar from the analysed diagram, but TrB and LB oils were separated from the others by the lower content of estragole. Methyl eugenol was dominant in essential oils of RB, WB and TrB, while contents of eugenol, trans-caryophyllene, β-elemene and germacrene were variable among these oils. The volatiles were also categorised according to their chemical structure types (Figure 1b). It was apparent that the majority of the VOCs was of phenylpropanoids, followed by the sesquiterpene hydrocarbon and oxygenated terpenes. The free volatiles of the monoterpene were detected only in TrB and TB oils.

### 3.2. Attractiveness

The basil essential oils were able to attract ORF differently, and the vast majority of responsive ORF was male, as shown in Table 3. Although the efficiency of the essential oil was less than commercial ME, the essential oils of the RB (~25 flies) and WB (~19 flies) were comparable to the ME (28 flies). However, the numbers of responses were low for LB, TB, and TrB oils. 

### 3.3. Insect Olfactory Chemosensory Responses

Overall, the ORFs of both sexes responded to the volatile substances by giving more movement frequency of the antenna and forelegs than when treated with ethanol (Table 4). The highest response was with ME, following by the WB, TB, and RB, respectively, and the degree of response was stronger in male ORFs than from the female ORFs. By looking only at the male ORFs, the antennae movement was more frequent when applied with the essential oils than the ME, but a high frequency of foreleg movement was observed with the ME (Supplementary Materials Video S1). These, however, were less obvious in the female ORFs.

**Experiment 2: Paste matrix complexation with basil essential oil encapsulation**

From the first experiment, we found that the essential oils that were effective in attracting ORF were of the holy basils of red and white types, in which the finding was in correspondent with its high content of methyl eugenol. In addition, the percentage yield of these essential oil types was considerably higher (~1%) compared with other basil types. Considering the material availability and yield, we then selected white holy basil oil (WBO) for the substantial studies. 

### 3.4. Emulsion Characteristic and Encapsulation Characteristics 

The viscosity of different wall material pastes is illustrated in Table 5. The result showed that the paste solution of GA gave high viscosity of 2976 cP. However, when mixed with MD, the viscosities decreased to 1348.0, 631.0, and 422.0, with 75%, 50%, and 25% MD content, respectively. 

After loading with WBO, the loading capacity and encapsulation efficiency were also shown in the same table. The GA complexation gave a higher loading capacity (~0.3 µL/0.2 g) than that of the absolute MD. In addition, the GA-consisting products also gave the high WBO encapsulation efficiency. The 25:75 ratio of MD:GA had the highest efficiency of containing WBO at ~9.4%.

The SEM images illustrate surface characteristics of each processed capsule (Table 6). The WBO loaded products exhibited tiny pore characteristics on the surface complexation. 

### 3.5. FTIR

**Figure 2:**
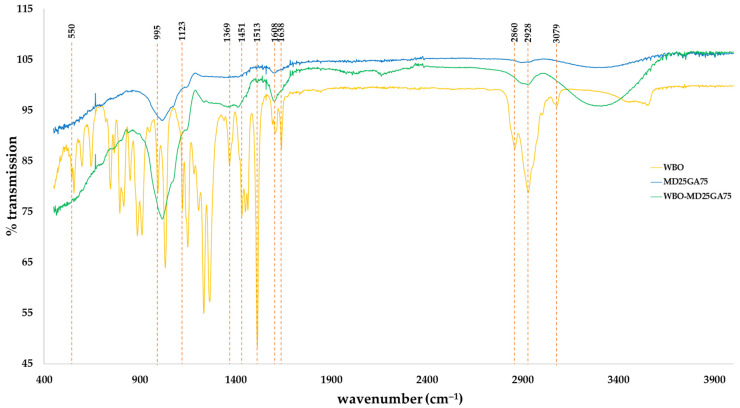
FTIR spectra of white holy basil oil (WBO), the wall material (MD25GA75), and white holy basil oil-loaded product (WBO-MD25GA75).

The FTIR patterns of the WBO-encapsulated products and wall material (MD25GA75) are illustrated in Figure 2. We were interested mainly in the FTIR characteristics of holy basil essential oil previously described by Sutaphanit and Chitprasert [50] and Agatonovic-Kustrin et al. [51]. The C–H bending peaks of the alkene/aromatic groups were detected in the FTIR spectra at ~550 and ~995 cm^−1^, and at ~1123 cm^−1^ represented C–O stretching vibrations. The spectrum at ~1369 and ~1451 cm^−1^ was assigned to the symmetric and asymmetric C–H bending vibrations in –CH_3_, respectively. The bands observed at ~1513, ~1608, and ~1638 cm^−1^ indicated the –C=C– stretching vibrations of alkene/aromatic, symmetric and asymmetric C–H stretching vibrations in –CH_2_– are present in the bands at ~2860 and ~2928 cm^−1^ and asymmetric C–H stretching vibration in =CH_2_ at the band of ~3079 cm^−1^. The FTIR spectrum peaks of WBO-encapsulated product showed evidence of the functional groups of those detected in WBO marked at ~995, ~1369, ~1451, ~1608, and ~2928 cm^−1^. This implied that WBO was successfully loaded and remained in the wall material. It is also worth highlighting that there is no significant chemical interaction between WBO and the wall material based on the detected spectra. 

### 3.6. Controlled Release of the White Holy Basil-Encapsulate Product

**Figure 3:**
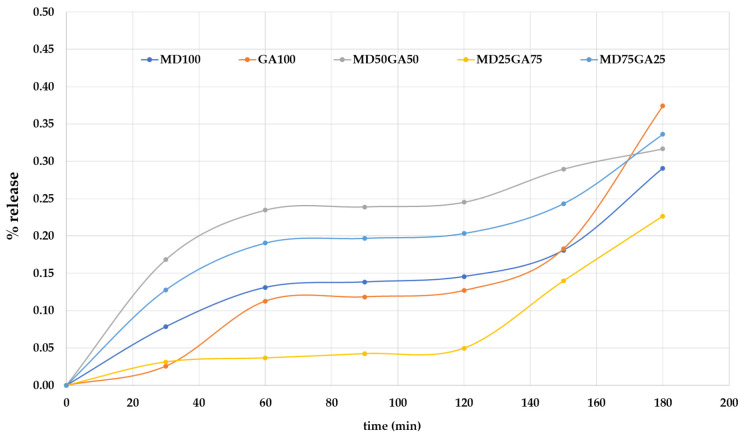
The percentage of volatile release as a function of time.

The volatile release characteristic of encapsulated products was given as in Figure 3. The results were reported according to the percentage of weight loss after exposure in the ambient condition. The releasing of all capsules during the first 120 min was initially subtle. However, after 120 min, capsules containing high GA (GA100 and MD25GA75) gave a remarkable increase in the release. In contrast, high doses of MD-containing capsules remained relatively stable.

**Experiment 3: WBO-encapsulated products efficacy in the field test study**

### 3.7. Product Efficacy 

The essential oil capsules portrayed different attraction capacities at different times (Table 7). At the initial 30 min, MD75GA25 attracted the most fruit flies among all other products and increased proportionately thereafter. The WBO encapsulated in the MD25GA75 formula was able to trap flies constantly (at about 0.25 files per trap) at intervals of 30–120 min and increased after 24 and 72 h exposure in the field. As for the GA100 product, the capacity was approximately 0.25 and 0.50 per trap at 24 and 72 h, respectively. Whereas WBO alone resulted in a rapid loss of the volatile active ingredients to the environment, thereby reducing the attractant ability. 

**Figure 4:**
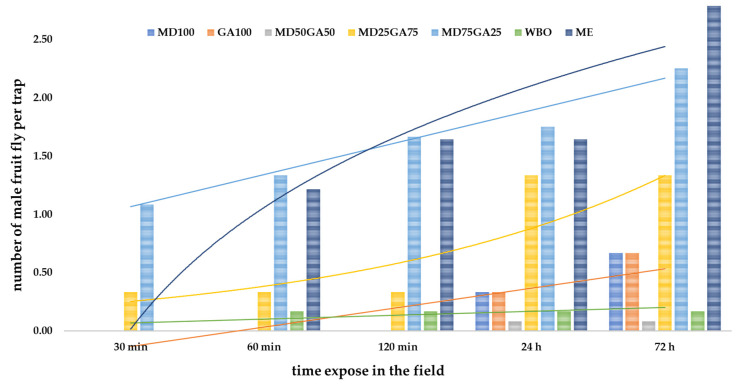
The trend of fruit flies caught by different lures at different times of field exposure.

The efficiency of the capsules combined with the fruit fly traps was by potting response curves of the efficacy data (Figure 4). The results showed that the encapsulation by paste method could progressively increase fruit fly attraction at later hours, especially in formulations with a mixture of GA and MD. Meanwhile, the product’s efficiency of the commercial ME trended to decrease over time.

## 4. Discussion

The volatile compositions from the essential oils of all basil types comprised the phenylpropanoid group, including methyl chavicol (estragole), eugenol and methyl eugenol. The others belong to the sesquiterpenes and monoterpenes. These compounds contribute to the unique sweet-anise aroma, which is the aroma characteristic of the essential oils from these species [28]. Moreover, the influence of gene expression for the biosynthesis of the phenylpropanoids is able to confer variation of the chemotypes within the same species. Thus, the altered levels between methyl chavicol (estragole) and linalool can be used to classified subspecies of the sweet basil [28,52,53,54]. Similarly, the constituents of the eugenol and methyl eugenol can be used to discriminate sub-varieties of the holy basil (*O.* *sanctum*) [30]. This is in agreement with our finding where the distinct levels of these phenylpropanoids were observed in both WB and RB. In addition, the content of methyl eugenol also depended upon the cultivation climate in which the intensity was with the *Ocimum* spp. grown in sub-tropical areas of North Africa, Eastern Europe, and parts of Asia [32,55]. Methyl eugenol is the primary volatile active ingredient in holy basils and is also known as a highly potent attractant for the ORF and other species in the Tephritidae family [56]. Male ORFs are attracted to this substance because it acts as a hormonal precursor and booster of male ORF’s sex pheromone [16,57]. Insect antennae is an essential organ in the olfactory system that escorts insect behavioural expression such as food hunting, mating, and finding appropriate sites for laying eggs [58]. First, the semiochemicals are detected by sensilla on the antennae, bound to its transmembrane odorant receptors (ORs) that generate the chemical signals transmitted into nerve impulses to the brain and control movement organs, such as forelegs [58,59]. In our study, insects responded to the VOCs with the increase in the movement frequency of the antennae and forelegs. The antennae’s higher frequency move was with the essential oils indicating the sensitivity of natural VOCs to its olfactory organs. Gomez-Diaz, Martin, Garcia-Fernandez, and Alcorta [58], Leal [60] described that the insect sensilla comprises pores in the cuticle that allow odorants to diffuse into the nerve system. The located ORs are also described as odour-specific, especially natural products such as essential oils [61,62]. In the ORF, in particular, its ORs are able to bind structurally with all portions of methyl eugenol molecule and respond to ortho-substituted benzenes with adjacent oxygen atoms or isoelectronic equivalents [63]. Our study conveyed that male *B. dorsalis* responded to various forms of VOCs in the essential oils. However, only methyl eugenol composition rendered the movement of the forelegs towards the odorant sources. 

The WBO was encapsulated to improve the volatility of the active compounds. Maltodextrin and gum arabic are affordable polysaccharides that are often used in encapsulations [64,65]. Maltodextrin is a water-soluble substance of low viscosity, low sugar content and free from colour and smell [66]. Likewise, GA is an emulsifier and enveloping material [67]. The viscosity of the MD was less but increased thereafter mixing with the GA. The efficiency of WBO loading also increased from the higher GA ratio in wall material formulation. Akhavan Mahdavi et al. [68] suggested that the encapsulation properties of the compounds were improved when the encapsulation mixture was composed of more than one type of wall material. The spectrum patterns were able to characterise the functional groups of the substance by the absorption or transmission, and the appearance of the vibration peak indicated [51]. The FTIR and SEM results indicated that the encapsulation characteristics of the wall materials were structurally formed. This was, however, not a chemical bond encapsulation as the volatile organics were not soluble in the water-soluble wall materials. The finding was in line with Sutaphanit and Chitprasert [50], in which the holy basil oil was not fully dissolved in the gelatine wall. 

Our field study was conducted during the sunlight to test the ability of the product as an attractant and the efficiency compared to the methyl eugenol. The temporal pattern of the mating behaviour of fruit flies: courtship episodes of the flies were usually between 0–9 h, and the striking peak of the activity was before noon [69,70]. The decision of female flies to mate with male fruit flies depends on long-range attraction signals, willingness to copulate, and male-male competition [71]. On the other hand, males decide to mate upon their libido and external stimuli, such as methyl eugenol [72]. Courtship usually takes place on fruits and fruit trees, and the estimated male success rate of mating is only around 0.6 per female per day [70]. Without mechanical traps, delivery of methyl eugenol fumigation enhances the mating success of *B. carambolae* and *B. dorsalis* males [73], which is not recommended for the non-sterile insect approach. Therefore, it is crucial to maintain the release of VOCs with semiochemical properties during the courtship episodes to be successful in the mechanical trap. Our finding demonstrated that paste encapsulation with MD combined with GA could maintain the release rate of the essential oil. 

## Figures and Tables

**Table 1:** Wall material types and ratio.

Treatments	Ratio	Emblem
Maltodextrin (MD)	100	MD100
Gum Arabic (GA)	100	GA100
MD–GA	50–50	MD50GA50
MD–GA	75–25	MD75GA25
MD–GA	25–75	MD25GA75

**Table 2:** Chemical compositions of the essential oils from five *Ocimum* spp. plants.

No.	RI	Compounds	Volatile Types	Amount of Chemical (µg/mL Essential Oils ± SE)				
				LB	RB	TB	TrB	WB
1	785	methyl 2-methylbutanoate	others	nd	nd	nd	2.56 ± 0.07	nd
2	818	(E)-hex-2-enal	others	nd	nd	nd	1.04 ± 0.12	nd
3	974	β-pinene	monoterpene	nd	nd	2.72 ± 0.06	nd	nd
4	976	1-octen-3-ol	others	3.64 ± 0.12	nd	nd	7.58 ± 0.27	nd
5	981	6-methyl-5-hepten-2-one	others	11.02 ± 0.41	nd	nd	2.25 ± 0.08	nd
6	987	myrcene	monoterpene	nd	nd	3.15 ± 0.11	7.89 ± 0.21	nd
7	991	6-methyl-5-Hepten-2-ol	others	1.25 ± 0.06	nd	nd	nd	nd
8	996	3-octanol	others	nd	nd	nd	4.29 ± 0.14	nd
9	1127	l-limonene	monoterpene	nd	nd	2.23 ± 0.08	nd	nd
10	1129	1,8-cineole	monoterpenoid	2.41 ± 0.09	nd	37.91 ± 1.28	nd	nd
11	1136	trans-ocimene	monoterpene	nd	nd	nd	169.23 ± 4.57	nd
12	1144	(E)-3,7-dimethylocta-1,3,6-triene	monoterpene	nd	nd	9.42 ± 0.34	nd	nd
13	1144	ocimene	monoterpene	nd	nd	nd	7.98 ± 0.26	nd
14	1185	fenchone	monoterpenoid	2.64 ± 0.09	nd	nd	nd	nd
15	1198	linalool	monoterpenoid	32.64 ± 1.33	6.95 ± 0.21	1.77 ± 0.07	45.01 ± 1.90	7.41 ± 0.41
16	1201	6-methyl-hepta-3,5-dien-2-one	others	nd	nd	nd	2.00 ± 0.12	nd
17	1219	(3E,5E)-2,6-dimethyl-1,3,5,7-octatetraene	monoterpene	nd	nd	nd	2.42 ± 0.06	nd
18	1226	2,6-dimethyl-2,4,6-octatriene	monoterpene	nd	nd	nd	20.29 ± 0.51	nd
19	1237	trans-epoxyocimene	monoterpenoid	nd	nd	nd	0.89 ± 0.05	nd
20	1242	camphor	monoterpenoid	5.48 ± 0.22	nd	13.57 ± 0.57	nd	3.25 ± 0.13
21	1249	nerol oxide	monoterpenoid	2.01 ± 0.11	nd	nd	nd	nd
22	1267	l-borneol	monoterpenoid	nd	10.50 ± 0.32	nd	nd	11.99 ±0.42
23	1268	p-Mentha-1,5-dien-8-ol	monoterpenoid	nd	nd	nd	2.13 ± 0.10	nd
24	1292	fenchol	monoterpenoid	13.05 ± 0.30	nd	1.48 ± 0.04	nd	3.65 ± 0.10
25	1294	estragole	phenylpropanoid	316.87 ± 15.12	14.98 ± 0.47	609.47 ± 20.00	nd	84.17 ± 2.81
26	1299	(E,E)-2,6-dimethyl-3,5,7-octatrien-2-ol	monoterpenoid	nd	nd	nd	10.96 ±0.43	nd
27	1323	nerol	monoterpenoid	15.35 ± 0.66	nd	nd	nd	nd
28	1335	neral	monoterpenoid	13.43 ± 0.55	nd	nd	nd	nd
29	1349	geraniol	monoterpenoid	9.67 ± 0.35	nd	nd	nd	nd
30	1365	geranial	monoterpenoid	18.74 ± 0.76	nd	nd	nd	nd
31	1380	bornyl acetate	others	2.48 ± 0.10	nd	nd	nd	nd
32	1447	eugenol	phenylpropanoid	3.15 ± 0.12	48.84 ± 1.28	nd	224.24 ± 9.97	83.04 ± 2.79
33	1471	α-copaene	sesquiterpene	3.57 ± 0.18	24.67 ± 0.13	nd	25.06 ± 1.15	8.86 ± 0.31
34	1478	β-elemene	sesquiterpene	9.38 ± 0.35	119.06 ± 3.99	nd	2.97 ± 0.19	68.52 ± 2.89
35	1479	β-bourbonene	sesquiterpene	nd	nd	nd	2.78 ±0.13	nd
36	1497	methyl eugenol	phenylpropanoid	28.12 ± 1.08	335.58 ± 11.99	9.57 ± 1.30	1.11 ± 0.01	372.57 ± 9.49
37	1509	rotundene	sesquiterpene	nd	1.48 ± 0.02	nd	nd	nd
38	1515	trans-caryophyllene	sesquiterpene	51.20 ± 2.17	193.96 ± 6.54	nd	19.11 ± 0.95	99.38 ± 3.20
39	1532	trans-α-bergamotene	sesquiterpene	27.55 ± 1.18	nd	10.91 ± 0.61	83.64 ± 3.58	10.08 ±0.38
40	1553	α-humulene	sesquiterpene	18.49 ± 0.76	14.82 ± 0.41	nd	2.68 ± 0.30	9.95 ± 0.38
41	1581	germacrene	sesquiterpene	13.31 ± 0.54	60.35 ± 2.12	2.83 ± 0.15	37.65 ± 1.77	25.13 ± 0.87
42	1585	trans-β-farnesene	sesquiterpene	2.71 ± 0.11	nd	nd	7.40 ± 0.39	nd
43	1589	β-selinene	sesquiterpene	3.53 ± 0.13	7.18 ± 0.20	nd	nd	6.21 ± 0.26
44	1596	(Z,E)-α-farnesene	sesquiterpene	nd	nd	nd	24.55 ±1.03	nd
45	1596	α-selinene	sesquiterpene	2.96 ± 0.12	13.85 ± 0.33	nd	nd	7.79 ± 0.39
46	1603	α-bulnesene	sesquiterpene	nd	nd	1.06 ± 0.01	nd	1.46 ± 0.06
47	1611	trans-α-bisabolene	sesquiterpene	2.89 ± 0.12	nd	nd	0.68 ± 0.03	nd
48	1614	β-copaene	sesquiterpene	5.66 ± 0.27	nd	4.97 ± 0.28	nd	4.28 ± 0.17
49	1619	δ-cadinene	sesquiterpene	3.31 ± 0.13	9.37 ± 0.20	nd	7.21 ± 0.37	5.39 ± 0.25
50	1625	β-sesquiphellandrene	sesquiterpene	nd	nd	nd	5.49 ±0.30	nd
51	1641	cis-α-bisabolene	sesquiterpene	37.24 ± 1.47	nd	nd	nd	2.06 ± 0.07
52	1647	elemol	sesquiterpenoid	nd	2.94 ± 0.02	nd	nd	2.30 ± 0.19
53	1671	spathulenol	sesquiterpenoid	nd	nd	1.36 ±0.03	3.27 ±0.16	nd
54	1676	caryophyllene oxide	sesquiterpenoid	30.16 ± 1.09	21.31 ± 0.44	nd	13.26 ± 0.65	15.71 ± 0.61
55	1682	lanceol	sesquiterpenoid	nd	nd	nd	1.58 ± 0.02	nd
56	1686	salvial-4(14)-en-1-one	sesquiterpenoid	nd	nd	nd	2.63 ± 0.27	nd
57	1801	humulene epoxide II	sesquiterpenoid	7.94 ± 0.29	nd	2.11 ± 0.00	nd	nd
58	1808	cubenol	sesquiterpenoid	nd	nd	2.21 ± 0.14	nd	nd
59	1809	ledene oxide-(II)	sesquiterpenoid	nd	nd	nd	3.99 ± 0.04	nd
60	1835	τ-cadinol	sesquiterpenoid	3.28 ± 0.11	nd	14.08 ± 0.79	nd	3.45 ± 0.16
61	1842	8-methylene-dispiro [2.1.2.4] undecane	others	1.78 ± 0.04	nd	nd	nd	nd
62	1847	β-eudesmol	sesquiterpenoid	1.25 ± 0.03	nd	nd	nd	nd
63	1849	α-cadinol	sesquiterpenoid	nd	nd	nd	2.99 ± 0.05	nd
64	1850	juniper camphor	sesquiterpenoid	nd	5.73 ± 1.50	nd	nd	2.59 ± 0.12
65	1880	α-bisabolol	sesquiterpenoid	2.37 ± 0.07	nd	nd	nd	nd
Percent yield essential oils from dried plant materials				0.86 ± 0.32	1.02 ± 0.21	1.13 ± 0.32	0.26 ± 0.02	1.04 ± 0.06
Compounds detected				36	17	18	35	23
Monoterpene hydrocarbons (%)				0.0	0.0	2.4	27.4	0.0
Oxygenated monoterpene (%)				16.2	2.0	7.5	7.8	3.1
Sesquiterpene hydrocarbons (%)				25.6	49.9	2.7	28.9	29.7
Oxygenated sesquiterpenes (%)				6.3	3.4	2.7	3.7	2.9
Others (%)				51.8	44.8	84.7	32.3	64.3

LB; lemon basil (*O. citriodorum*), RB; red holy basil (*O. sanctum* var. Rama), TB; Thai basil (*O. basilicum* var. thyrsiflorum), TrB; tree basil (*O. gratissimum*), and WB; white holy basil (*O. sanctum* var. Shyama). Values are calculated as a reference to the internal standard toluene (0.015 mg/mL), 3 replications, SE = standard error, nd = not detected, RI = Retention Indices determined concerning a homologous series of n-alkanes on a Rxi-5Sil MS column.

**Table 3:** The attractiveness of five essential oils and commercial fruit fly attractant to Oriental fruit flies.

Volatile Organic Compounds	Average of Number of Trapped Insects		
	Treatment (*n* ± SE/100)		Control (*n* ± SE/100)
LB	5.00 ± 1.68 a		0
	95.00%♂	5.00%♀	
RB	25.00 ± 2.48 bc		0.5 ± 0.25
	90.00%♂	10.00%♀	
TB	4.00 ± 1.47 a		0.25 ± 0.22
	93.75%♂	6.25%♀	
TrB	2.00 ± 1.41 a		0.25 ± 0.22
	100.00%♂	0.00%♀	
WB	18.75 ± 2.69 b		0
	94.66%♂	5.44%♀	
ME	28.75 ± 2.87 c		0.5 ± 0.25
	89.56%♂	10.44%♀	

LB; lemon basil (*O. citriodorum*), RB; red holy basil (*O. sanctum* var. Rama), TB; Thai basil (*O. basilicum* var. thyrsiflorum), TrB; tree basil (*O. gratissimum*), WB; white holy basil (*O. sanctum* var. Shyama) and ME; commercial fruit fly attractant (methyl eugenol). The mean difference in the treatment column was analysed using one-way ANOVA, and the letters (a–c) indicated the significant difference at 95 confidence level. SE = standard error.

**Table 4:** The olfactory chemosensory responses of Oriental fruit flies to volatile organic compounds and different basil essential oils.

Volatile Organic Compounds	Frequency of Movement			
	Male		Female	
	Antennae	Forelegs	Antennae	Forelegs
LB	+	++	++	++
RB	+++	+++	+++	+++
TB	+++	++++	+++	+++
TrB	++	+	++	+
WB	++++	++++	++	+++
ME	++	+++++	++	++
Ethanol	+	++	+	+++

+ level of frequent movement, LB; lemon basil (*O. citriodorum*), RB; red holy basil (*O. sanctum* var. Rama), TB; Thai basil (*O. basilicum* var. thyrsiflorum), TrB; tree basil (*O. gratissimum*), WB; white holy basil (*O. sanctum* var. Shyama), and ME; commercial fruit fly attractant (methyl eugenol).

**Table 5:** Characteristics of the wall materials.

Wall Materials	Emulsion		WBO-Encapsulated Products	
	Viscosity (cP)	Stability (%Separation)	Oil Loading (µL/0.2 g) *	Encapsulation Efficiency (%)
MD100	150.0	>1	0.163 ± 0.0218 a	4.27
GA100	2976.0	-	0.303 ± 0.0362 ab	7.92
MD50GA50	631.0	-	0.313 ± 0.0728 ab	8.18
MD25GA75	1348.0	-	0.360 ± 0.0678 b	9.39
MD75GA25	422.0	>1	0.235 ± 0.0493 ab	6.14

* The difference of mean ± SE (standard error) of three replications in the oil loading column was analysed using one-way ANOVA. The letters (a,b) indicated the significant difference at 95 confidence level.

**Table 6:** The capsule structures.

The Structure of Processed Maltodextrin and Gum Arabic	
MD100	GA100
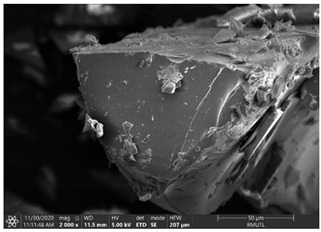	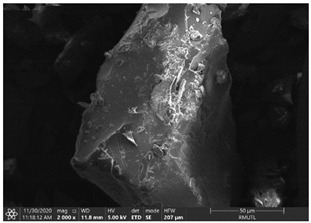
**The Structure of White Holy Basil Essential Oil (WBO) Loaded Capsules**	
WBO-MD100	WBO-GA100
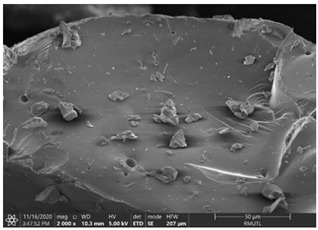	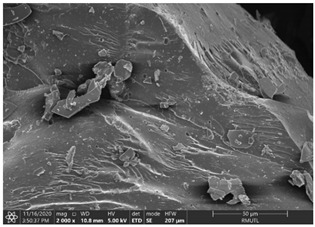
WBO-MD50GA50	WBO-MD25GA75
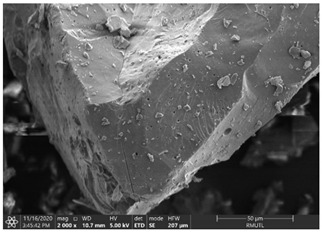	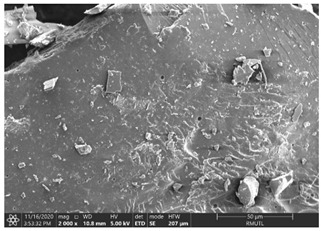
WBO-MD75GA25	
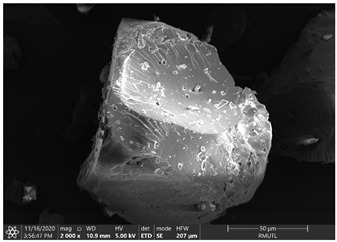	

**Table 7:** Number of flies per trap with different lures at the period of 72 h.

Product Formulas (Treatments)	The Average Number of Oriental Fruit Fly (Fly/Trap)				
	Time of Exposure in the Field				
	Mins			h	
	30	60	120	24	72
MD100	0.00 ± 0.00 a	0.00 ± 0.00 a	0.00 ± 0.00 a	0.25 ± 0.46 a	0.75 ± 0.89 b
GA100	0.00 ± 0.00 a	0.00 ± 0.00 a	0.00 ± 0.00 a	0.25 ± 0.46 a	0.50 ± 0.92 ab
MD50GA50	0.00 ± 0.00 a	0.00 ± 0.00 a	0.00 ± 0.00 a	0.31 ± 0.33 a	0.31 ± 0.33 a
MD25GA75	0.25 ± 0.46 ab	0.25 ± 0.46 a	0.25 ± 0.46 a	1.00 ± 0.75 bc	1.00 ± 0.75 b
MD75GA25	0.75 ± 1.39 b	1.00 ± 1.30 b	1.25 ± 1.16 b	1.50 ± 0.92 c	2.00 ± 0.00 c
White holy basil essential oil	0.00 ± 0.00 a	0.25 ± 0.46 a	0.25 ± 0.46 a	0.50 ± 0.53 b	0.50 ± 0.53 ab
Control (CTR)	0.00 ± 0.00	0.00 ± 0.00	0.00 ± 0.00	0.00 ± 0.00	0.00 ± 0.00

The difference of mean ± SE (standard error) of eight replications in the same column was analysed by one-way ANOVA and the letters (a–c) are indicated the significantly different at 95 confidence level.

## Data Availability

Not applicable.

## Supplementary Materials

The following are available online at https://zenodo.org/record/5094021#.YO0VZEwRVPZ, Video S1: the olfactory chemosensory responses of male Oriental fruit fly with a drop of the following VOCs; ethanol (at 0.30 min), methyl eugenol (at 1.39 min), ethanol (at 3.05 min), white holy basil oil (at 4.10 min), ethanol (at 5.20 min), methyl eugenol (at 6.30 min), ethanol (at 7.36 min), and white holy basil oil (at 8.36 min).
